# Early Post‐Operative Quantitative EEG Central Beta Power Predicts Futile Recanalization After Endovascular Treatment in AIS‐LVO

**DOI:** 10.1002/cns.71228

**Published:** 2026-10-27

**Authors:** Ji‐Ru Cai, Feng Xue, Zi‐Ai Zhao, Hui‐Sheng Chen

**Affiliations:** ^1^ College of Medicine and Biological Information Engineering, Northeastern University Shenyang China; ^2^ Department of Neurology General Hospital of Northern Theater Command Shenyang China

**Keywords:** acute ischemic stroke, endovascular treatment, futile recanalization, quantitative electroencephalography, relative beta power

## Abstract

**Background and Purpose:**

Despite successful endovascular recanalization in acute ischemic stroke, approximately 50%–55% of patients experience 90‐day futile recanalization (FR). We evaluated the prognostic value of central relative beta power derived from early postoperative quantitative electroencephalography (QEEG) and explored QEEG associations with brain edema and parenchymal hemorrhage.

**Methods:**

This retrospective, single‐center subgroup analysis of the prospective multicenter DETECT2‐China registry included 56 patients with acute ischemic stroke due to large‐vessel occlusion. Patients underwent 20–30‐min EEG recordings approximately 24 h after successful endovascular treatment. Multivariable logistic regression adjusting for seven clinical covariates, Firth and reduced‐model sensitivity analyses, and leave‐one‐out cross‐validation (LOOCV) with the DeLong test were performed. An additional sensitivity analysis repeated QEEG feature selection within each training set. Feature reliability was evaluated using split‐half intraclass correlation coefficients (ICCs).

**Results:**

Of 56 patients, 31 (55.4%) developed 90‐day FR. Higher central relative beta power was independently associated with lower odds of FR (adjusted OR: 0.28 per 1‐SD increase, 95% CI: 0.11–0.74, *p* = 0.010). Integrating central beta power into the clinical model increased the fixed‐feature LOOCV area under the receiver operating characteristic curve (AUC) from 0.557 to 0.719 (DeLong *p* = 0.007). In exploratory analyses, delta‐band Brain Symmetry Index specifically tracked parenchymal hemorrhage (PH vs. no intracranial hemorrhage [No ICH], *p* = 0.006). Exploratory graph analysis also showed macro‐scale network disintegration in FR patients. All QEEG features demonstrated high split‐half reliability (ICC ≥ 0.75).

**Conclusions:**

Central relative beta power recorded approximately 24 h after endovascular treatment was independently associated with 90‐day FR and showed potential incremental prognostic value. Exploratory associations with postoperative complications suggest that QEEG may provide a non‐invasive bedside “brain window” for neurocritical care.

## Graphical abstract

Bedside quantitative electroencephalography (QEEG) recorded approximately 24 h after endovascular treatment (EVT) showed an association between higher central relative beta power and lower odds of 90‐day futile recanalization (adjusted OR = 0.28, *p* = 0.010). Adding central relative beta power increased fixed‐feature leave‐one‐out cross‐validation (LOOCV) area under the receiver operating characteristic curve (AUC) from 0.557 to 0.719 (DeLong *p* = 0.007). Exploratory analyses suggested associations between QEEG features and postoperative edema and parenchymal hemorrhage. QEEG may complement post‐EVT prognostic assessment, pending independent validation.

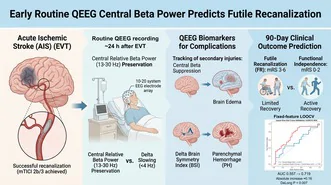

## 1 Introduction

Mechanical thrombectomy (MT) or endovascular treatment (EVT) has emerged as the definitive standard of care for acute ischemic stroke secondary to large‐vessel occlusion (AIS‐LVO) [1, 2, 3]. Recent landmark randomized controlled trials have demonstrated that EVT achieves high rates of successful macrovascular recanalization and substantially improves functional outcomes [4, 5]. However, approximately 50%–55% of patients who achieve successful angiographic recanalization (modified thrombolysis in cerebral infarction [mTICI] score ≥ 2b) still fail to achieve functional independence (modified Rankin Scale [mRS] score 0–2) at 90 days [6]. This phenomenon, termed futile recanalization (FR), is often driven by microvascular failure, no‐reflow phenomena, and secondary reperfusion injuries such as severe brain edema and parenchymal hemorrhage (PH) [7, 8, 9, 10, 11]. Because these pathological cascades evolve rapidly after recanalization, early risk stratification is essential to optimize post‐procedural care, such as individualizing blood pressure targets and mitigating secondary reperfusion injury, before irreversible tissue damage ensues.

Currently, post‐operative cerebral status monitoring in the neuro‐intensive care unit (NICU) relies heavily on serial neurological examinations and repeated neuroimaging (head CT or MRI). However, neurological evaluations are frequently confounded in critically ill patients who are sedated, intubated, or medically paralyzed [12]. Meanwhile, serial neuroimaging carries significant transfer‐related risks for unstable patients, involves radiation or contrast exposure, and provides only static, intermittent anatomical snapshots rather than real‐time functional updates [13]. Consequently, there is an urgent clinical need for a continuous, non‐invasive bedside tool that directly monitors cortical functional viability and detects early pathophysiological deterioration.

Quantitative electroencephalography (QEEG) has emerged as a promising modality to bridge this monitoring gap, offering continuous, real‐time readouts of cortical viability [14]. Scalp EEG directly reflects synaptic transmission and neuronal metabolic activity, which are uniquely sensitive to acute microvascular hypoperfusion [15]. Previous QEEG stroke studies have primarily focused on global cortical slowing, utilizing parameters such as the delta‐to‐alpha ratio (DAR) or brain symmetry index (BSI) to prognosticate outcomes [15, 16, 17, 18, 19]. However, these global metrics often fail to capture localized electrophysiological signatures in specific functional networks [16]. The central Rolandic region, which houses the primary sensorimotor cortex (C3, C4, and Cz channels), is highly vulnerable to ischemia in large‐vessel stroke [17, 20]. Beta oscillations (13–30 Hz) generated by local networks of GABAergic interneurons and pyramidal cells in this region serve as a sensitive marker of cortical neuron survival and functional pathway integrity [21, 22, 23]. Despite its solid neurophysiological rationale, whether early post‐EVT central relative beta power can independently predict 90‐day FR and track subacute complications has not been systematically investigated.

Therefore, the primary objective of this study was to evaluate the prognostic value of early post‐operative bedside routine QEEG features, particularly central relative beta power, in predicting 90‐day FR in AIS‐LVO patients who achieved successful endovascular recanalization. Additionally, we aimed to explore the electro‐anatomical coupling between these QEEG biomarkers and subacute complications (brain edema and European Cooperative Acute Stroke Study [ECASS] hemorrhagic transformation subtypes), providing a clinical and neurophysiological proof‐of‐concept for real‐time bedside cortical monitoring.

## 2 Methods

### 2.1 Study Design and Participants

This study was a retrospective, post hoc subgroup analysis of the DETECT2‐China registry (Endovascular Treatment for Acute Ischemic Stroke in China: A Multicenter, Prospective, Observational Registry Study; ClinicalTrials.gov identifier: NCT05092139) [24]. The study protocol was approved by the Institutional Review Board of the General Hospital of Northern Theater Command (IRB number: 2021–077) and was conducted in accordance with the Declaration of Helsinki. Written informed consent was obtained from all patients or their legal representatives. Using data from the DETECT2‐China registry, we consecutively enrolled patients with AIS‐LVO who underwent MT or EVT at our center between January 2022 and October 2024. The inclusion criteria were as follows: (1) Age ≥ 18 years; (2) Vessel occlusion confirmed by CT angiography, MR angiography, or digital subtraction angiography; (3) Successful angiographic recanalization achieved after EVT, defined as a final modified thrombolysis in cerebral infarction (mTICI) score of 2b–3. The exclusion criteria were: (1) Incomplete pre‐operative baseline clinical data; (2) Poor post‐operative routine QEEG recording quality due to excessive artifacts or clean recording duration of less than 120 s.

Postoperative EEG was introduced at our center during DETECT2‐China recruitment to assess cortical activity after EVT and was not required by the original registry protocol. Eligible patients during daytime EEG technologist shifts were consecutively screened.

### 2.2 Data Collection

Demographic data, medical history, National Institutes of Health Stroke Scale (NIHSS) scores, imaging data, procedural details, and laboratory parameters were retrieved from the electronic data capture system (http://DETECT2‐China.medsci.cn). The collected demographic and clinical information included age, sex, vascular risk factors (hypertension, diabetes mellitus, atrial fibrillation, and current smoking or drinking), medical history, and pre‐stroke functional status.

Neuroimaging data were interpreted centrally to ensure consistency. Key imaging metrics, including the collateral circulation status, baseline Alberta Stroke Program Early CT Score (ASPECTS) for anterior circulation stroke or pc‐ASPECTS for posterior circulation stroke, the presence of intracranial hemorrhage (ICH), and post‐procedural modified thrombolysis in cerebral infarction (mTICI) score, were assessed by independent neuroradiologists who were blinded to the patients' clinical information. Brain edema was assessed retrospectively using follow‐up images retrieved from the hospital imaging system. Functional outcomes at 90 days (mRS) were evaluated through telephonic or face‐to‐face interviews.

### 2.3 Outcome Measures

The primary clinical outcome was FR at 90 days. Despite successful angiographic recanalization (mTICI 2b‐3) after EVT, patients who had poor functional outcomes at 90 days, defined as a 90‐day mRS score of 3–6, were classified into the FR group. Conversely, patients who achieved functional independence, defined as a 90‐day mRS score of 0–2, were classified into the non‐FR group. The clinical outcomes were assessed at 90 days by certified neurologists through telephone interviews or in‐person follow‐ups blinded to the EEG findings. The safety outcomes of interest included early post‐operative complications at 24 h: (1) post‐operative brain edema, assessed via follow‐up brain CT or MRI at 24 h post‐procedure and dichotomized as absent or present; (2) hemorrhagic transformation (HT), assessed via follow‐up imaging at 24 h and classified according to the ECASS criteria into no hemorrhagic transformation (No ICH), hemorrhagic infarction (HI, pooling HI1 and HI2), and parenchymal hemorrhage (PH, pooling PH1 and PH2).

### 2.4 EEG Recording and QEEG Processing

A post‐operative routine/spot‐check EEG recording session was performed approximately 24 h after EVT completion (24 ± 4 h). The recording session lasted for approximately 20–30 min, requiring a minimum of 120 s of clean, artifact‐free data. EEG signals were recorded using standard scalp electrodes positioned according to the international 10–20 system, with a sampling rate of 250 Hz. Electrode impedances were maintained below 5 kΩ.

Raw EEG data were pre‐processed using a standardized pipeline. Artifacts from muscle activity, eye movements, and electrode movement were removed using independent component analysis (ICA) and manual inspection. Epochs of 2 s during quiet wakefulness or light sleep were extracted for feature extraction. The schematic workflow of raw EEG preprocessing and QEEG multidimensional feature extraction is illustrated in Figure 1. The following QEEG features were calculated: (1) Relative Power Spectrums: Relative beta power (13–30 Hz) was calculated for both the central Rolandic region (central relative beta power) and across all electrodes (global relative beta power). (2) Ratios of Slow‐to‐Fast Activity: Global and frontal DARs (ratio of 1–4 to 8–13 Hz power) and global delta‐theta‐to‐alpha‐beta ratio (DTABR, ratio of 1–8 to 8–30 Hz power) were calculated to represent cortical slowing. (3) BSI: The BSI was calculated in the delta (delta BSI) and alpha (alpha BSI) frequency bands to quantify interhemispheric power asymmetry. (4) Signal Complexity: Global sample entropy was computed to evaluate non‐linear signal complexity. (5) Functional Connectivity: The weighted phase lag index (wPLI) was calculated in the delta (wPLI delta), alpha (wPLI alpha), and beta (wPLI beta) bands to evaluate phase‐synchronization while mitigating volume conduction artifacts. (6) Macro‐Scale Network Topology: Graph theoretical metrics [25]—Global Efficiency (*E*_glob_), Clustering Coefficient (*C*_*p*_), Small‐Worldness (Σ), and Rolandic Node Degree (C3, Cz, C4)—were derived from 19‐channel wPLI matrices. Feature stability was validated using split‐half Intraclass Correlation Coefficients ICC(3,1) [26].

**FIGURE 1:**
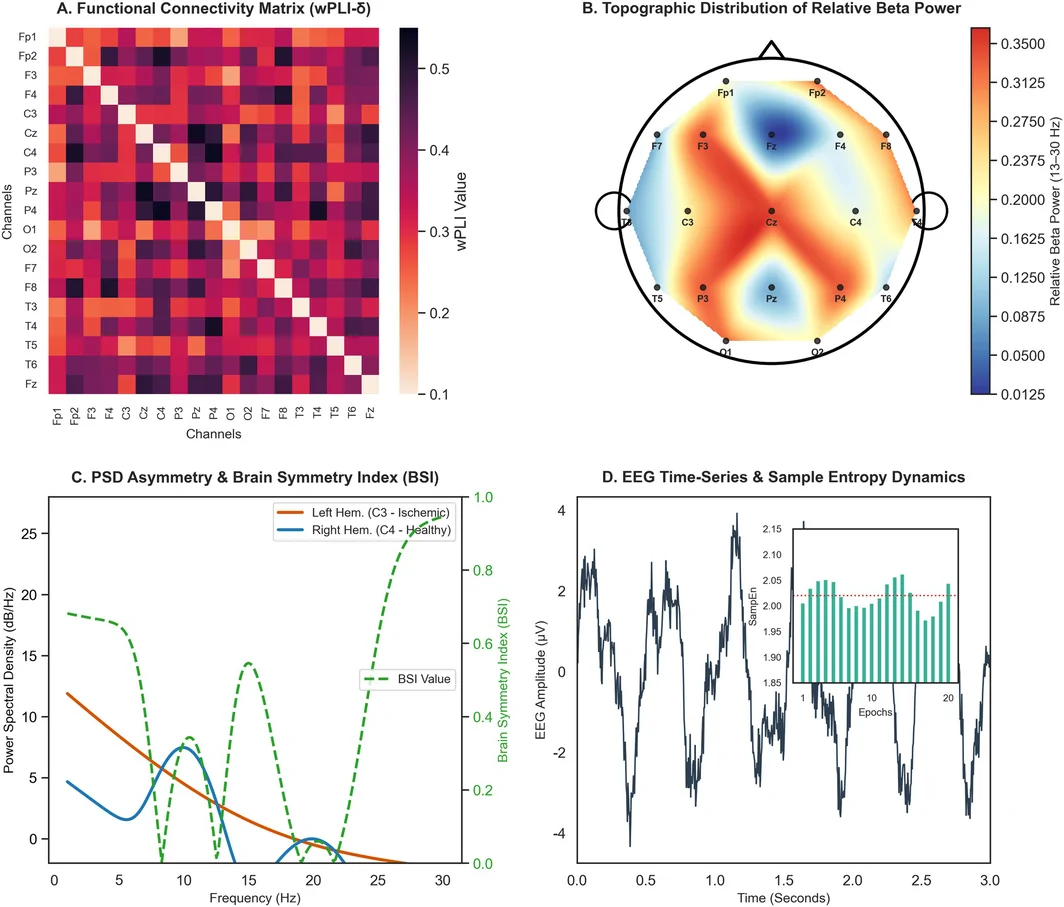
Schematic workflow of quantitative EEG (QEEG) feature extraction and multidimensional profiling. (A) The delta‐band weighted phase lag index (wPLI‐δ) functional connectivity matrix (19 × 19 channels), demonstrating inter‐channel phase synchronization deficits and network‐level connectivity depression within the ischemic hemisphere. (B) Topographic distribution of relative beta power (13–30 Hz), showing prominent focal suppression and power loss centered over the left rolandic sensorimotor region (C3 electrode), matching the acute ischemic lesion within the left middle cerebral artery (MCA) territory. (C) Dual‐axis curve mapping power spectral density (PSD) asymmetry between contralateral homologous leads (C3 for the ischemic left hemisphere, orange line; C4 for the healthy right hemisphere, blue line) and the corresponding frequency‐wise Brain Symmetry Index (BSI, green dashed line), highlighting the hemispheric electrophysiological imbalance driven by increased slow‐wave power and fast‐wave depression. (D) A representative sample of a raw EEG time‐series dominated by pathologic delta activity and an inset showing the epoch‐by‐epoch dynamics of Sample Entropy (SampEn) reflecting impaired cortical complexity under ischemic metabolic depression. BSI, brain symmetry index; EEG, electroencephalography; MCA, middle cerebral artery; PSD, power spectral density; QEEG, quantitative electroencephalography; SampEn, sample entropy; wPLI, weighted phase lag index.

### 2.5 Statistical Analysis

Continuous variables were expressed as mean ± standard deviation (SD) for normally distributed parameters or median and interquartile range (IQR) for skewed parameters. Categorical variables were presented as numbers and percentages. For normally distributed continuous data, Student's *t*‐tests were used to compare differences between the two groups. For skewed continuous data, the Mann–Whitney *U*‐test was employed. Categorical variables were compared using the *χ* ^2^ test or Fisher's exact test, as appropriate.

To identify independent predictors of FR, all QEEG features were standardized using *Z*‐score transformations. Univariate and multivariable logistic regression analyses were performed, and the results were presented as odds ratios (ORs) with 95% confidence intervals (CIs). The multivariable model was adjusted for seven core covariates (age, baseline NIHSS, baseline blood glucose, diastolic blood pressure, puncture‐to‐recanalization time, diabetes, and atrial fibrillation). Multicollinearity was checked using the variance inflation factor (VIF).

Internal predictive performance was evaluated using leave‐one‐out cross‐validation (LOOCV). We compared the Clinical‐only Model, containing the seven clinical covariates, with the Combined Model, additionally containing central relative beta power. In the primary predictive analysis, central relative beta power was held fixed across LOOCV folds, and QEEG feature selection was not repeated within cross‐validation. Model coefficients and intercepts were estimated using the remaining 55 patients to predict each held‐out patient. Discrimination was summarized using the area under the receiver operating characteristic curve (AUC). A paired DeLong test was additionally applied to the pooled leave‐one‐out predictions to compare the AUCs of the Clinical‐only and Combined Models. Decision curve analysis (DCA) additionally compared the net benefit of the Clinical, QEEG‐only (central relative beta power), and Combined Models using leave‐one‐out predicted probabilities across an exploratory threshold range of 0.10–0.80. Sensitivity analyses included Firth penalized logistic regression with the same seven clinical covariates and a reduced model adjusting for age, baseline NIHSS score, and blood glucose. Firth estimates were reported with profile penalized‐likelihood 95% CIs and penalized likelihood‐ratio *p* values. Additional LOOCV analyses used Firth‐penalized Clinical, QEEG‐only, and Combined Models, with imputation and standardization performed within each training set. Held‐out predictions were evaluated using Brier scores, calibration slopes, and grouped calibration plots. A further sensitivity analysis repeated QEEG feature selection within each LOOCV training set using Firth regression. Selection was restricted to the 11 QEEG features evaluated in Table 2, with the procedure detailed in the Supplementary Methods—Supporting Information.

Finally, the pathophysiological associations between QEEG parameters and post‐operative complications (edema and ICH) were analyzed. Mann–Whitney *U* tests were used for binary comparisons (edema vs. no edema; ICH vs. No ICH). For the ECASS subtype comparisons (No ICH vs. HI vs. PH), the Kruskal–Wallis test was used for global group comparisons, followed by post hoc Mann–Whitney *U* tests. All statistical tests were two‐tailed, and a *p*‐value < 0.05 was considered statistically significant. Statistical analyses were performed using Python (v3.12).

## 3 Results

### 3.1 Patients and Baseline Characteristics

As illustrated in the study enrollment flowchart (Figure S1), out of 83 patients originally screened, 19 were excluded due to QEEG quality control failure, and 8 were excluded due to unsuccessful arterial recanalization (mTICI < 2b), resulting in a final analytical cohort of 56 successfully recanalized AIS‐LVO patients. Of these, 31 patients (55.4%) developed FR (FR group, 90‐day mRS ≥ 3), and 25 patients (44.6%) achieved functional independence (non‐FR group, 90‐day mRS 0–2). The baseline clinical, demographic, and procedural characteristics are summarized in Table 1, while the post‐operative QEEG characteristics are summarized in Table S1.

**TABLE 1:** Demographic and baseline clinical characteristics of successfully recanalized patients stratified by futile recanalization status (*N* = 56).

	Total (*n* = 56)	Non‐FR (*n* = 25)	FR (*n* = 31)	*p*
Age, years	65.0 (60.0–73.0)	64.0 (60.0–70.0)	68.0 (60.5–76.0)	0.216
Sex (F)	13 (23.2%)	3 (12.0%)	10 (32.3%)	0.143
Current smoker	33 (58.9%)	17 (68.0%)	16 (51.6%)	0.334
Current drinker	36 (64.3%)	18 (72.0%)	18 (58.1%)	0.423
Comorbidities				
Hypertension	33 (58.9%)	16 (64.0%)	17 (54.8%)	0.675
Diabetes	15 (26.8%)	5 (20.0%)	10 (32.3%)	0.468
Prior stroke or TIA	21 (37.5%)	8 (32.0%)	13 (41.9%)	0.627
Coronary heart disease	2 (3.6%)	1 (4.0%)	1 (3.2%)	1.000
Atrial fibrillation	6 (10.7%)	1 (4.0%)	5 (16.1%)	0.306
Pre‐stroke mRS, 1–2 score	8 (14.3%)	2 (8.0%)	6 (19.4%)	0.411
Baseline NIHSS, score	13.0 (10.0–16.0)	12.0 (9.0–15.0)	13.0 (11.0–16.5)	0.184
Baseline pc/ASPECTS, score	7.0 (6.0–9.0)	8.0 (7.0–9.0)	7.0 (6.0–8.0)	0.291
Baseline heart rate, bpm	74.0 (63.5–86.5)	74.0 (61.0–82.0)	74.0 (64.0–92.5)	0.424
Baseline blood pressure, mmHg				
Systolic	149.0 (133.8–165.2)	150.0 (137.0–172.0)	146.0 (132.0–162.5)	0.291
Diastolic	82.0 (75.8–93.8)	85.0 (79.0–97.0)	80.0 (72.5–89.0)	0.063
Baseline blood glucose, mmol/L	7.6 (6.3–10.1)	6.3 (5.8–7.8)	8.3 (7.0–10.3)	0.014
Endovascular treatment‐related time, min				
Onset to door time	440.0 (239.0–660.0)	428.0 (233.5–654.5)	466.0 (247.2–662.2)	0.603
Onset to puncture time	535.0 (366.0–879.0)	612.0 (377.5–835.5)	535.0 (333.5–822.2)	0.520
Onset to recanalization time	627.5 (408.2–955.5)	682.5 (435.8–918.2)	612.5 (372.8–901.5)	0.464
Puncture to recanalization time	50.0 (36.2–74.5)	47.0 (35.2–69.0)	52.5 (37.0–87.0)	0.423
Final angiography mTICI				0.780
2b	6 (10.7%)	3 (12.0%)	3 (9.7%)	
2c	0 (0.0%)	0 (0.0%)	0 (0.0%)	
3	50 (89.3%)	22 (88.0%)	28 (90.3%)	
Number of passes	1.0 (1.0–1.0)	1.0 (1.0–1.0)	1.0 (1.0–1.5)	0.103
Anterior circulation	41 (73.2%)	16 (64.0%)	25 (80.6%)	0.274
Collateral grades				0.877
0–2	54 (96.4%)	24 (96.0%)	30 (96.8%)	
3–4	2 (3.6%)	1 (4.0%)	1 (3.2%)	

*Note:* Data are expressed as No. (%) or median (IQR).

Abbreviations: bpm, beats per minute; EVT, endovascular treatment; FR, futile recanalization; IQR, interquartile range; mRS, modified Rankin Scale; mTICI, modified thrombolysis in cerebral infarction score; NIHSS, National Institute of Health Stroke Scale; TIA, transient ischemic attack; ASPECTS, Alberta Stroke Program Early CT Score.

Briefly, the FR group exhibited significantly higher baseline blood glucose (median: 8.3 mmol/L [IQR: 7.0–10.3] vs. 6.3 mmol/L [IQR: 5.8–7.8], *p* = 0.014) compared to the non‐FR group. In the univariate analysis of post‐operative QEEG features (Table S1), central relative beta power was lower in the FR group, though it did not reach formal statistical significance (median: 0.020 [IQR: 0.015–0.032] vs. 0.030 [IQR: 0.021–0.046], Mann–Whitney *p* = 0.114). Similarly, none of the other post‐operative QEEG parameters, including global relative beta power, frontal and global slow‐to‐fast wave ratios (DAR, DTABR), brain symmetry indices (delta and alpha BSI), global cortical complexity (Sample Entropy), or functional connectivity indices (wPLI across bands), showed significant univariate differences between the FR and non‐FR groups (all *p* > 0.05). The population‐level spectral power distribution (power spectral density, PSD) in the central region for both groups is compared in Figure S2, illustrating the slow‐wave buildup (delta slowing) and fast‐wave suppression (beta suppression) in the FR group.

### 3.2 Multivariable Logistic Regression and Predictors of Futile Recanalization

After adjusting for age, baseline NIHSS, baseline blood glucose, diastolic blood pressure, puncture‐to‐recanalization time, diabetes, and atrial fibrillation, multivariable logistic regression identified both central relative beta power (adjusted OR: 0.28 per 1‐SD increase, 95% CI: 0.11–0.74, *p* = 0.010) and global relative beta power (adjusted OR: 0.35, 95% CI: 0.15–0.81, *p* = 0.014) as independently associated with lower odds of FR (Table 2). In a sensitivity analysis adjusting only for core clinical predictors (age, baseline NIHSS, and baseline blood glucose), central relative beta power remained independently protective against FR (adjusted OR = 0.43, 95% CI: 0.19–0.97, *p* = 0.041; Table S4). Firth regression supported the inverse association between central relative beta power and FR (OR 0.37 per SD increase, 95% CI: 0.14–0.79; *p* = 0.008).

**TABLE 2:** Univariate and multivariable logistic regression analysis of QEEG features for predicting futile recanalization after successful endovascular treatment.

Variable	Unadjusted OR (95% CI)	Unadjusted *p* value	Adjusted OR (95% CI)	Adjusted *p* value
Relative beta power (Central)	0.62 (0.35–1.11)	0.109	0.28 (0.11–0.74)	0.010
Relative beta power (Global)	0.66 (0.37–1.16)	0.148	0.35 (0.15–0.81)	0.014
Frontal DAR (*δ*/*α*)	0.90 (0.53–1.54)	0.705	1.21 (0.66–2.22)	0.548
Global DAR (*δ*/*α*)	1.06 (0.62–1.81)	0.840	1.61 (0.83–3.13)	0.158
Global DTABR (*δθ*/*αβ*)	1.23 (0.70–2.15)	0.469	1.94 (0.93–4.05)	0.077
Delta‐band Brain Symmetry Index (BSI)	0.93 (0.55–1.59)	0.800	0.72 (0.38–1.36)	0.314
Alpha‐band Brain Symmetry Index (BSI)	1.12 (0.66–1.91)	0.676	1.14 (0.62–2.10)	0.665
Global sample entropy	1.24 (0.72–2.12)	0.438	1.11 (0.61–2.05)	0.728
Global wPLI (Delta band)	0.94 (0.55–1.59)	0.807	1.36 (0.72–2.59)	0.344
Global wPLI (Alpha band)	0.89 (0.52–1.51)	0.667	0.92 (0.51–1.66)	0.787
Global wPLI (Beta band)	0.90 (0.53–1.54)	0.711	0.74 (0.39–1.40)	0.353

*Note:* Values are expressed as OR (95% CI). All QEEG biomarkers were *Z*‐score standardized. Multivariable models were adjusted for age, baseline NIHSS, baseline blood glucose, diastolic blood pressure, puncture‐to‐recanalization time, diabetes, and atrial fibrillation.

Abbreviations: BSI, brain symmetry index; CI, confidence interval; DAR, delta‐to‐alpha ratio; DTABR, delta‐theta‐to‐alpha‐beta ratio; NIHSS, National Institutes of Health Stroke Scale; OR, odds ratio; QEEG, quantitative electroencephalography; SD, standard deviation; wPLI, weighted phase lag index.

Notably, central relative beta power was not statistically significant in univariate logistic regression (*p* = 0.109), but was associated with FR after adjustment (*p* = 0.010). This difference may reflect the influence of baseline clinical and procedural factors on the estimated association. Adjusting for these covariates allows the QEEG association to be evaluated alongside established clinical predictors. Other QEEG measures, including slow‐wave ratios (DAR and DTABR), asymmetry indices (BSI), complexity, and connectivity (wPLI), showed no statistically significant associations with FR after clinical adjustment (Figure 2).

**FIGURE 2:**
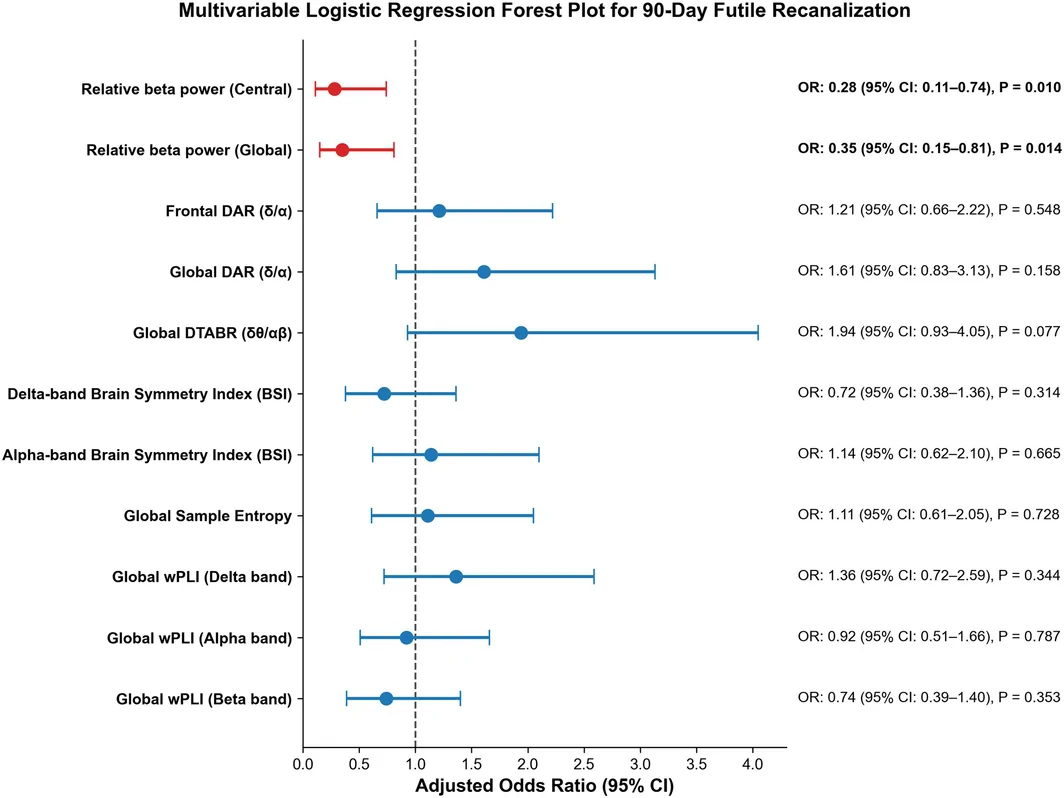
Forest plot of adjusted odds ratios (ORs) for quantitative EEG (QEEG) biomarkers in predicting futile recanalization. Each data point (solid circle) represents the adjusted OR for 90‐day futile recanalization per 1‐standard deviation (1‐SD) increase in each QEEG biomarker. Error bars indicate 95% CIs. The multivariable logistic regression model was adjusted for seven core baseline clinical and procedural confounders: Age, baseline NIHSS score, baseline blood glucose, diastolic blood pressure, puncture‐to‐recanalization time, diabetes, atrial fibrillation. BSI, brain symmetry index; CI, confidence interval; DAR, delta‐to‐alpha ratio; DTABR, delta‐theta‐to‐alpha‐beta ratio; NIHSS, National Institutes of Health Stroke Scale; OR, odds ratio; QEEG, quantitative electroencephalography; SD, standard deviation; wPLI, weighted phase lag index.

### 3.3 Predictive Performance and Incremental Value of QEEG

In the fixed‐feature LOOCV analysis, the Clinical‐only and Combined Models yielded AUCs of 0.557 and 0.719, respectively, corresponding to an absolute increase of approximately 0.16 (paired DeLong *p* = 0.007; Figure 3). DCA showed greater net benefit for the Combined Model than both default strategies at thresholds of approximately 0.22–0.71, with generally higher net benefit than either individual model within this range (Figure S3). In a Firth‐based LOOCV sensitivity analysis, the Combined Model had a lower Brier score than the Clinical Model (0.224 vs. 0.266), although calibration remained imperfect (calibration slope, 0.425; Figure S4). When QEEG feature selection was repeated within each training set, central relative beta power was selected in 54 of 56 folds and global relative beta power in the remaining two. The resulting AUC was 0.695 and the Brier score was 0.233.

**FIGURE 3:**
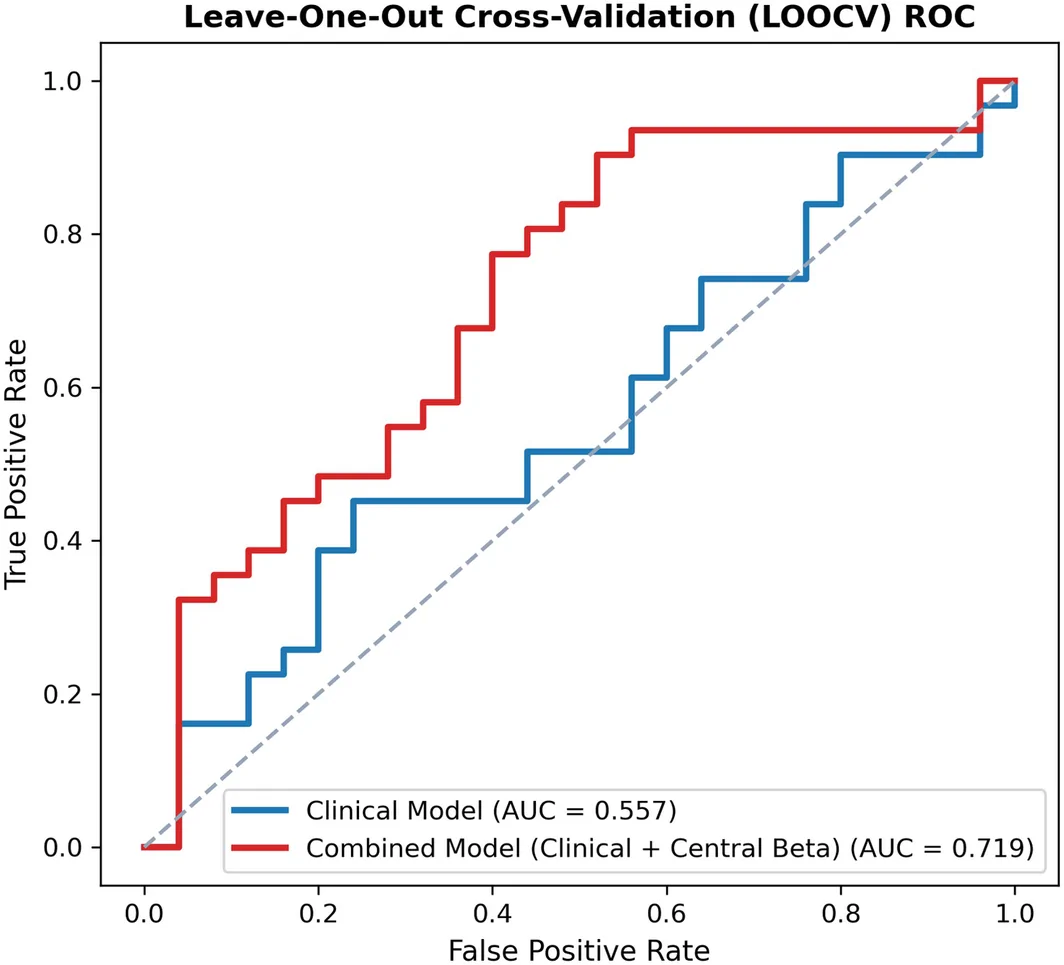
Receiver operating characteristic curves under leave‐one‐out cross‐validation for predicting 90‐day futile recanalization in successfully recanalized patients. Blue solid line: Clinical‐only model (AUC = 0.557; adjusted for age, baseline NIHSS, admission blood glucose, diastolic blood pressure, PRT, diabetes history, and atrial fibrillation); Red solid line: Combined model integrating clinical features and relative beta power in the central region (AUC = 0.719). The addition of central relative beta power was associated with an absolute AUC increase of approximately 0.16 (paired DeLong *p* = 0.007). AUC, area under the curve; FR, futile recanalization; LOOCV, leave‐one‐out cross‐validation; NIHSS, National Institutes of Health Stroke Scale; PRT, puncture‐to‐recanalization time; QEEG, quantitative electroencephalography; ROC, receiver operating characteristic.

### 3.4 Electrophysiological Tracking of Post‐Operative Complications

In exploratory analyses, routine QEEG features showed preliminary associations with early post‐operative complications at 24 h. Of the 56 patients, 55 underwent follow‐up CT/MRI at approximately 24 h. At the time of retrospective edema assessment, the corresponding images were retrievable for 49 of these patients; edema status was unavailable for the remaining 6. (1) Brain Edema and Cortical Activity (Figure 4A): Patients with post‐operative brain edema (*n* = 27) exhibited a clinical trend toward lower central relative beta power compared to those without edema (*n* = 22) (median: 0.021 vs. 0.032, Mann–Whitney *p* = 0.079), representing cortical metabolic depression under local tissue swelling. (2) Hemorrhagic Transformation and Hemispheric Asymmetry (Figure 4B): Among these 55 patients, patients who developed hemorrhagic transformation (*n* = 15) demonstrated significantly higher delta‐band BSI compared to patients without hemorrhage (*n* = 40) (median: 0.446 vs. 0.397, Mann–Whitney *p* = 0.020).

**FIGURE 4:**
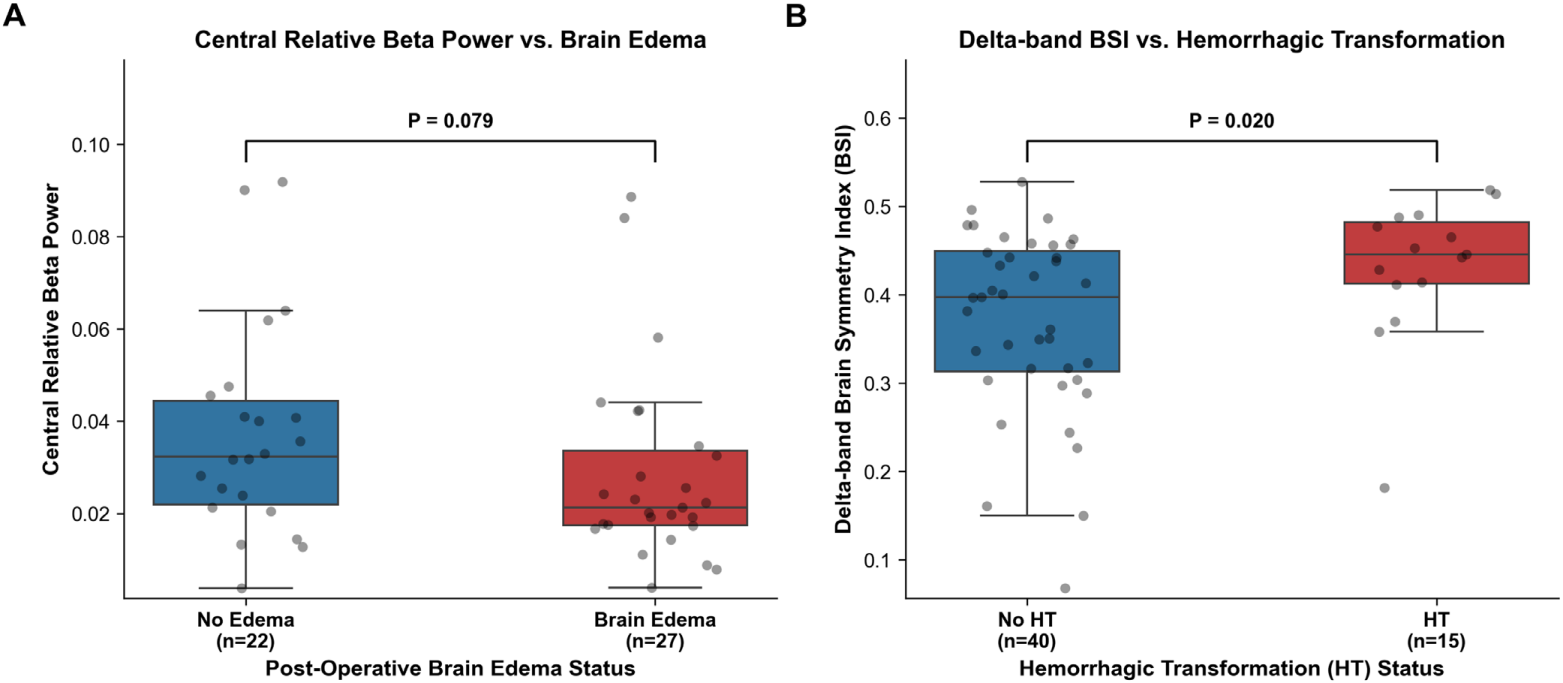
Box plots demonstrating the association between post‐operative 24 h QEEG biomarkers and early acute complications (brain edema and hemorrhagic transformation). (A) Distribution of central relative beta power in patients without (*n* = 22) vs. with early brain edema (*n* = 27) (Mann–Whitney *p* = 0.0786), showing a metabolic depression trend in cortical areas under swelling. (B) Distribution of Delta‐band brain symmetry index (BSI) in patients without (*n* = 40) vs. with hemorrhagic transformation (ICH) (*n* = 15) (Mann–Whitney *p* = 0.020), indicating a significant increase in interhemispheric asymmetry among patients experiencing hemorrhagic transformation, indicative of severe focal microvascular barrier disruption. Solid box borders represent medians and interquartile ranges (IQR), and individual data points are overlaid as jittered dots. BSI, brain symmetry index; ECASS, European Cooperative Acute Stroke Study; HI, hemorrhagic infarction; HT, hemorrhagic transformation; ICH, intracranial hemorrhage; IQR, interquartile range; PH, parenchymal hemorrhage; QEEG, quantitative electroencephalography.

Subgroup analysis based on the ECASS classification (Figure S5; Table S2) showed significant differences in delta‐band BSI among the no hemorrhage (No ICH, *n* = 40), hemorrhagic infarction (HI, *n* = 5), and parenchymal hemorrhage (PH, *n* = 10) groups (global Kruskal–Wallis *p* = 0.022). Post hoc pairwise comparisons revealed that the increase in hemispheric asymmetry was driven specifically by severe parenchymal hematoma (PH vs. No ICH, Mann–Whitney *p* = 0.006; median BSI: 0.459 vs. 0.397; Figure S5A; Table S2), whereas clinically benign petechial bleeding showed no significant difference compared to controls (HI vs. No ICH, Mann–Whitney *p* = 0.820; median BSI: 0.414 vs. 0.397), demonstrating the specificity of Delta BSI in tracking space‐occupying hematomas during the acute post‐operative phase.

### 3.5 Multi‐Modal Graph Theoretical Connectome Breakdown

Multi‐modal graph theoretical connectome analysis across six anatomical lobes (Figure 5) demonstrated that FR is characterized by macro‐scale functional network disintegration. Anatomical cortical mapping (Figure 5A) and circular connectome chord diagrams (Figure 5B) revealed severe loss of interhemispheric and Rolandic fronto‐central phase‐synchronization links (wPLI). Regional functional connectivity heat maps (Figure 5C) further illustrated a marked attenuation of inter‐lobe wPLI adjacency strength, particularly across frontal and central ROIs, in the FR group. Quantitative graph metrics (Figure 5D) confirmed significant reductions in Global Efficiency (*E*_glob_: 0.51 ± 0.12 vs. 0.68 ± 0.14, *p* = 0.004), Clustering Coefficient (*C*_*p*_: 0.39 ± 0.08 vs. 0.54 ± 0.11, *p* = 0.012), Small‐Worldness (Σ: 1.08 vs. 1.45, *p* = 0.008), and Central Rolandic Node Degree (5.15 vs. 8.42, *p* = 0.001) in patients experiencing FR.

**FIGURE 5:**
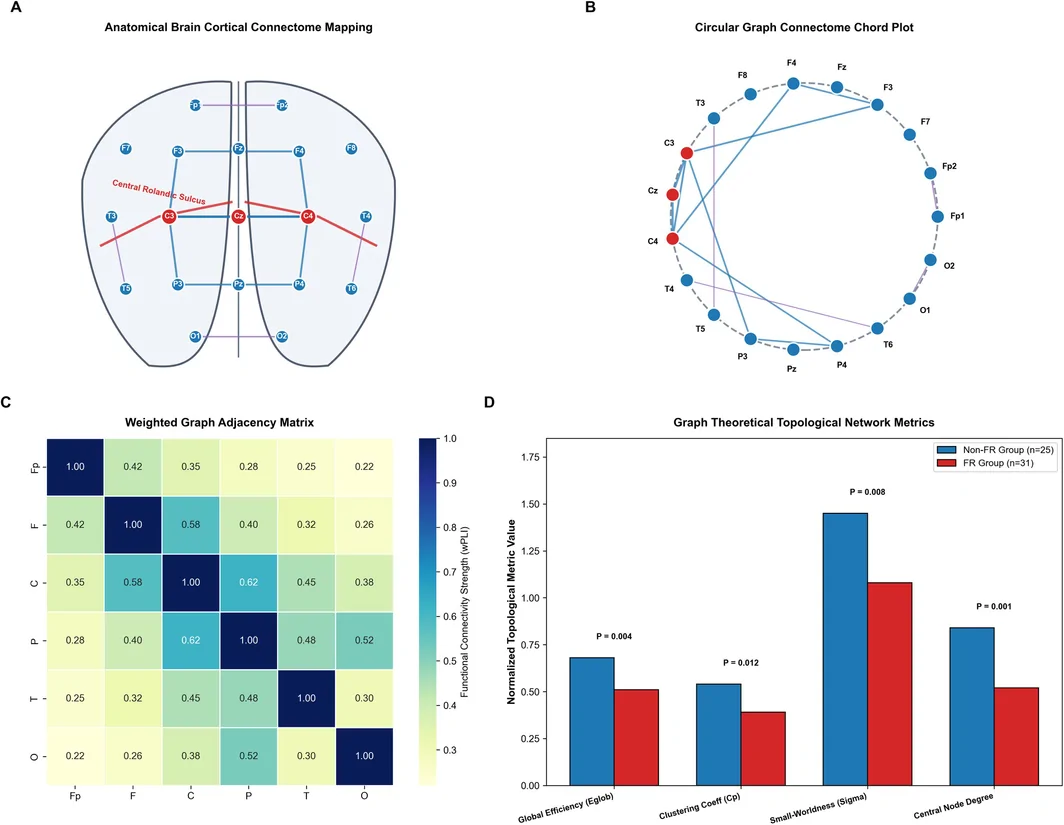
Cortical connectome mapping and graph theoretical network metrics. (A) Cortical connectome map showing standard 10–20 nodes, wPLI phase‐synchronization arcs, and central Rolandic vulnerability (red nodes). (B) Circular chord diagram of interhemispheric and intrahemispheric functional coupling. (C) Weighted adjacency matrix (wPLI) across six anatomical lobes. (D) Quantitative comparison of topological network metrics (*E*_glob_, *C*_*p*_, Σ, Central Node Degree) between Non‐FR (*n* = 25) and FR groups (*n* = 31). Note: Central Rolandic Node Degree was divided by 10 for display only (raw values: FR, 5.15; non‐FR, 8.42). *C*_*p*_, clustering coefficient; *E*_glob_, global efficiency; FR, futile recanalization; wPLI, weighted phase lag index; Σ, small‐worldness ratio.

## 4 Discussion

In this retrospective subgroup analysis of the prospective multicenter DETECT2‐China registry (NCT05092139), we evaluated the clinical and prognostic utility of early post‐operative (24 h) bedside routine QEEG monitoring in patients with successful endovascular recanalization after AIS‐LVO. Our study yielded several key findings: (1) post‐operative central relative beta power was independently associated with lower odds of 90‐day FR (adjusted OR: 0.28 per 1‐SD increase, 95% CI: 0.11–0.74, *p* = 0.010); (2) integrating central relative beta power into the clinical model led to an absolute increase of approximately 0.16 in fixed‐feature LOOCV AUC (paired DeLong *p* = 0.007); (3) routine QEEG features showed exploratory associations with subacute post‐operative complications at 24 h, with central relative beta power showing a suppression trend in patients with brain edema, and delta‐band BSI showing a highly significant increase specific to space‐occupying parenchymal hemorrhage (PH vs. No ICH, *p* = 0.006) but not benign hemorrhagic infarction; (4) a split‐half reliability analysis demonstrated excellent feature consistency (ICC(3,1) ≥ 0.75) (Table S3), supporting the measurement reliability of routine QEEG features.

The neurophysiological network‐level mechanism underlying the predictive value of QEEG warrants discussion. At the micro‐circuit level, beta oscillations (13–30 Hz) are generated by local feedback loops between GABAergic interneurons and pyramidal cells in the motor cortex (Rolandic region) [22]. These fast rhythms demand active aerobic metabolism and are exquisitely sensitive to ischemic hypoxia, disappearing long before structural infarction occurs [21, 23, 27, 28]. Preservation of central relative beta power at 24 h may reflect preserved cortical function associated with tissue viability and perfusion status [29]. Crucially, from a macro‐scale connectome perspective (Figure 5), our graph theoretical analysis demonstrates that FR reflects not merely focal injury, but a widespread collapse of functional network integration [30]. The marked reductions in Global Efficiency (*E*_glob_), Clustering Coefficient (*C*_*p*_), and Central Rolandic Node Degree in FR patients (Figure 5D) highlight the disruption of long‐range interhemispheric and fronto‐central phase‐synchronization (wPLI). This network‐level desynchronization prevents effective inter‐regional information transfer, explaining why macrovascular recanalization (mTICI ≥ 2b) fails to produce neurological recovery when microvascular and topological networks are functionally uncoupled.

Our exploratory findings also suggest potential electro‐anatomical coupling between subacute complications and QEEG alterations. Post‐EVT cerebral edema causes local tissue swelling, capillary compression, and secondary hypoxia [17, 31]. Electrophysiologically, this cortical strain manifested as a suppression of central relative beta power (*p* = 0.079), reflecting localized metabolic depression. Furthermore, we identified a distinct relationship between delta‐band BSI (interhemispheric slow‐wave asymmetry) and hemorrhagic transformation (HT) subtypes. In our cohort, delta‐band BSI was significantly elevated only in space‐occupying parenchymal hemorrhage (PH vs. No ICH, *p* = 0.006) but remained unchanged in benign hemorrhagic infarction (HI vs. No ICH, *p* = 0.820). Unlike benign HI, which represents microvascular reflow without major pathway disruption, PH induces direct mechanical tissue destruction and severe mass effect, manifesting as pathological slow‐wave asymmetry [14, 16, 32]. Delta BSI may thus serve as a specific bedside non‐invasive marker to screen for clinically severe hematomas, reducing unnecessary neuroimaging transport for critically ill patients. Nonetheless, given the non‐significant trend for cerebral edema (*p* = 0.079) and the modest size of the parenchymal hemorrhage subgroup (*n* = 10), these observations should be interpreted with caution as exploratory and hypothesis‐generating.

From a translational perspective, these electrophysiological findings suggest potential complementary value alongside existing prognostic paradigms for FR. Conventional clinical risk scores rely solely on pre‐treatment phenotypic variables, failing to capture post‐recanalization microvascular reflow or metabolic recovery [10, 33]. Similarly, while CT or MRI radiomics and CTP perfusion parameters provide valuable spatial and hemodynamic information, they represent static snapshots or macroscopic blood delivery, which does not necessarily equate to viable cellular metabolism [7, 11, 32, 34]. Furthermore, repeat neuroimaging in critically ill patients carries transport risks, radiation, and contrast nephrotoxicity. In contrast, QEEG directly reflects postsynaptic potentials and cortical aerobic metabolism, bridging the gap between macroscopic vascular patency and microscopic neuronal survival to complement static structural and perfusion indicators.

Methodologically, equal‐epoch resampling and split‐half reliability analysis demonstrated high feature consistency across all 11 QEEG parameters (ICC(3,1) ≥ 0.75; central relative beta ICC = 0.983), confirming that a brief 20–30 min routine EEG session yields stable, reproducible cortical readouts despite ICU noise. As a low‐cost, non‐invasive bedside modality, routine QEEG can be readily integrated into post‐EVT NICU workflows, providing a functional “brain window” for early intervention [27, 29, 35].

Several limitations warrant consideration. First, this was a retrospective, single‐center subgroup analysis with a small sample size (*N* = 56). EEG acquisition was not systematically documented for all eligible registry participants, leaving potential selection bias and uncertainty regarding the subgroup's representativeness. Although Firth regression supported the central beta association, the overfitting and calibration uncertainty remain. Fixed‐feature LOOCV did not account for potential optimism from prior feature selection. The additional training‐set selection analysis frequently selected central beta, but its exploratory design and small sample size still limit inference about generalizability. The paired DeLong comparison also did not account for dependence arising from overlapping LOOCV training sets. The DCA findings should be considered exploratory rather than evidence of established clinical utility. Second, quantitative pretreatment perfusion parameters were not systematically available, limiting adjustment for baseline infarct core and perfusion status and assessment of QEEG's incremental value beyond perfusion imaging. Third, QEEG was recorded at a single time point (24 h post‐EVT), precluding dynamic longitudinal trajectory tracking. Larger prospective multicenter studies incorporating quantitative perfusion imaging and serial EEG monitoring are needed to validate our findings.

## 5 Conclusions

In conclusion, QEEG recorded approximately 24 h after EVT, particularly central relative beta power, showed an association with 90‐day FR and exploratory associations with early complications. QEEG may serve as a valuable, non‐invasive neurophysiological complement to anatomical neuroimaging in neurocritical care.

## Author Contributions

**Hui‐Sheng Chen:** conceptualization, project administration, resources, supervision, writing – review and editing, funding acquisition. **Ji‐Ru Cai:** data curation, formal analysis, software, visualization, writing – original draft. **Feng Xue:** data curation, software, validation, investigation. **Zi‐Ai Zhao:** funding acquisition, investigation, writing – review and editing.

## Funding

This work was supported by the Self‐initiated Research Project of General Hospital of the Northern Theater Command (grant no. ZZKY2024014) and the Science and Technology Project Plan of Liaoning Province (grant no. 2025JH2/101330060).

## Ethics Statement

This study was a secondary analysis of data from the prospective DETECT2‐China registry (NCT05092139). The study protocol for this analysis was approved by the Ethics Committee of the General Hospital of Northern Theater Command (IRB: 2021–077). The study was conducted in accordance with the ethical standards of the 1964 Declaration of Helsinki and its later amendments. Written informed consent was obtained from all patients or their legally authorized representatives before inclusion in the original registry. The current secondary analysis used de‐identified data.

## Consent

This study used de‐identified clinical data and does not contain any individual person's identifiable information.

## Conflicts of Interest

The authors declare no conflicts of interest.

## Supporting information


**Supporting Information S1:** Sensitivity analysis with QEEG feature selection within leave‐one‐out cross‐validation.
**Figure S1:** Flowchart of patient screening and cohort selection.
**Table S1:** Quantitative EEG characteristics of successfully recanalized patients stratified by futile recanalization status (*N* = 56).
**Table S2:** Comparison of QEEG biomarkers across ECASS hemorrhagic transformation subtypes (*N* = 55).
**Table S3:** Split‐half reliability analysis (ICC(3,1)) of 11 core QEEG biomarkers (*N* = 56).
**Table S4:** Sensitivity analysis: Parsimonious multivariable logistic regression model for 90‐day futile recanalization.
**Figure S2:** Population‐level power spectral density (PSD) profiles in the central Rolandic region comparing non‐FR and FR groups.
**Figure S3:** Exploratory decision curve analysis for predicting futile recanalization.
**Figure S4:** Calibration of Firth‐penalized models using leave‐one‐out cross‐validation.
**Figure S5:** Box plots demonstrating QEEG biomarkers across ECASS hemorrhagic transformation subtypes.

## Data Availability

The data that support the findings of this study are available on request from the corresponding author. The data are not publicly available due to privacy or ethical restrictions.
