# Supplementary material for: Early Post‐Operative Quantitative EEG Central Beta Power Predicts Futile Recanalization After Endovascular Treatment in AIS‐LVO
Source: CNS Neurosci Ther. 2026 Oct 27;32(10):e71228. doi: 10.1002/cns.71228 (PMC13711485; doi:10.1002/cns.71228)
Supplement: Supplementary file 1 [file CNS-32-e71228-s001.docx]

# Supporting Information

**Supplementary Methods.** Sensitivity analysis with QEEG feature selection within leave-one-out cross-validation.

**Supplementary Tables S1–S4**

**Supplementary Figures S1–S5.**

# Supplementary Methods

**Sensitivity analysis with QEEG feature selection within LOOCV**

Within each LOOCV training set of 55 patients, each of the 11 QEEG features listed in Table 2 was entered separately into a Firth logistic regression model with the same seven clinical covariates. Imputation and standardization parameters were estimated from the training set only. The feature with the smallest penalized likelihood-ratio P value was selected, without a significance threshold, and its fitted model predicted the held-out patient. Repeating this procedure across all 56 patients yielded held-out probabilities for calculating AUC and Brier score. This selection rule was specified for the revision-stage sensitivity analysis and was not a reconstruction of the original feature-selection process.

# Supplementary Figures and Tables

**Supplementary Figure S1. Flowchart of patient screening and cohort selection.** From 83 AIS patients in the DETECT-2 registry undergoing EVT with routine EEG recorded approximately 24 h postoperatively, 19 were excluded for QEEG quality control failure and 8 for unsuccessful recanalization (mTICI < 2b). The final cohort (N = 56) was divided into non-futile recanalization (n = 25; 90-day mRS 0–2) and futile recanalization (n = 31; 90-day mRS 3–6).

*Abbreviations:* AIS, acute ischemic stroke; EEG, electroencephalography; EVT, endovascular treatment; mRS, modified Rankin Scale; mTICI, modified Treatment in Cerebral Ischemia; QEEG, quantitative electroencephalography.

**Supplementary Table S1. Quantitative EEG characteristics of successfully recanalized patients stratified by futile recanalization status (N = 56).**

| **QEEG Features** | **Total (N = 56)** | **Non-FR (n = 25)** | **FR (n = 31)** | **P Value** |
| --- | --- | --- | --- | --- |
| **Spectral Power Metrics** | | | | |
| Relative beta power (Central) | 0.02 (0.02-0.04) | 0.03 (0.02-0.04) | 0.02 (0.02-0.03) | 0.114 |
| Relative beta power (Global) | 0.35 (0.23-0.44) | 0.36 (0.26-0.47) | 0.33 (0.22-0.40) | 0.161 |
| Frontal DAR (δ/α) | 5.19 (4.51-6.40) | 5.11 (4.09-5.77) | 5.33 (4.69-7.24) | 0.419 |
| Global DAR (δ/α) | 5.17 (2.95-9.27) | 5.70 (2.97-7.78) | 4.81 (2.88-11.09) | 0.856 |
| Global DTABR (δθ/αβ) | 3.91 (2.67-6.33) | 4.55 (2.78-6.02) | 3.91 (2.64-8.59) | 0.869 |
| **Symmetry and Complexity** | | | | |
| Delta-band Brain Symmetry Index (BSI) | 0.41 (0.32-0.46) | 0.42 (0.34-0.46) | 0.41 (0.32-0.45) | 0.633 |
| Alpha-band Brain Symmetry Index (BSI) | 0.30 (0.26-0.35) | 0.30 (0.27-0.33) | 0.29 (0.24-0.35) | 0.843 |
| Global Sample Entropy | 2.10 (2.05-2.14) | 2.11 (2.04-2.15) | 2.09 (2.05-2.13) | 0.587 |
| **Functional Connectivity (wPLI)** | | | | |
| Global wPLI (Delta band) | 0.46 (0.45-0.48) | 0.46 (0.44-0.48) | 0.46 (0.45-0.47) | 0.934 |
| Global wPLI (Alpha band) | 0.40 (0.38-0.42) | 0.40 (0.37-0.43) | 0.39 (0.38-0.42) | 0.598 |
| Global wPLI (Beta band) | 0.22 (0.21-0.22) | 0.21 (0.21-0.22) | 0.22 (0.21-0.23) | 1.000 |

***Note****:* QEEG metrics are expressed as median (IQR).

***Abbreviations****:* QEEG, quantitative electroencephalography; DAR, delta-to-alpha ratio; DTABR, delta-theta-to-alpha-beta ratio; BSI, brain symmetry index; wPLI, weighted phase lag index; FR, futile recanalization; IQR, interquartile range.

**Supplementary Table S2. Comparison of QEEG biomarkers across ECASS hemorrhagic transformation subtypes (N = 55)**

| **ECASS Subtype** | **Sample Size (n)** | **Delta BSI Median (IQR)** | **Delta BSI Post-hoc *P*** | **Central Relative Beta Median (IQR)** | **Central Beta Post-hoc *P*** |
| --- | --- | --- | --- | --- | --- |
| No ICH | 40 (72.7%) | 0.397 (0.313, 0.450) | Reference | 0.024 (0.018, 0.041) | Reference |
| HI | 5 (9.1%) | 0.414 (0.358, 0.428) | 0.820 | 0.040 (0.023, 0.042) | 0.435 |
| PH | 10 (18.2%) | 0.459 (0.443, 0.485) | 0.006 | 0.026 (0.020, 0.035) | 0.945 |
| Global Test (Kruskal-Wallis) | N=55 | H = 7.618 | *P* = 0.022 | H = 0.881 | *P* = 0.644 |

*Note:* Values are presented as Median (IQR) or Number (Percentage). One patient with missing follow-up imaging was excluded (N = 55 evaluated). Group comparisons across ECASS subtypes (No ICH, HI, PH) were evaluated using Kruskal-Wallis test, followed by post-hoc Mann-Whitney U tests vs. No ICH.

*Abbreviations:* BSI, brain symmetry index; ECASS, European Cooperative Acute Stroke Study; HI, hemorrhagic infarction; ICH, intracranial hemorrhage; IQR, interquartile range; PH, parenchymal hemorrhage; QEEG, quantitative electroencephalography.

**Supplementary Table S3. Split-half reliability analysis (ICC(3,1)) of 11 core QEEG biomarkers (N = 56)**

| **QEEG Feature Name** | **ICC(3,1)** | **95% CI** | **F-Statistic** | ***P* Value** |
| --- | --- | --- | --- | --- |
| Relative beta power (Central) | 0.9831 | 0.971, 0.990 | 117.32 | <0.001 |
| Relative beta power (Global) | 0.9935 | 0.988, 0.996 | 306.45 | <0.001 |
| Frontal DAR (δ/α) | 0.9606 | 0.933, 0.977 | 49.78 | <0.001 |
| Global DAR (δ/α) | 0.9698 | 0.949, 0.982 | 65.12 | <0.001 |
| Global DTABR (δθ/αβ) | 0.9717 | 0.952, 0.984 | 69.84 | <0.001 |
| Delta-band Brain Symmetry Index (BSI) | 0.8894 | 0.816, 0.934 | 17.08 | <0.001 |
| Alpha-band Brain Symmetry Index (BSI) | 0.9712 | 0.950, 0.983 | 68.39 | <0.001 |
| Global Sample Entropy | 0.9539 | 0.922, 0.973 | 42.45 | <0.001 |
| Global wPLI (Delta band) | 0.7674 | 0.631, 0.858 | 7.6 | <0.001 |
| Global wPLI (Alpha band) | 0.9484 | 0.912, 0.970 | 37.76 | <0.001 |
| Global wPLI (Beta band) | 0.8247 | 0.718, 0.894 | 10.41 | <0.001 |

*Note:* Intraclass Correlation Coefficients (ICC(3,1)), 95% confidence intervals, F-statistics, and *P*-values were computed using a two-way mixed-effects model (consistency type) based on split-half resampling between odd and even 2-second epochs. ICC ≥ 0.75 represents excellent reliability.

*Abbreviations:* BSI, brain symmetry index; CI, confidence interval; DAR, delta/alpha ratio; DTABR, delta-theta/alpha-beta ratio; ICC, intraclass correlation coefficient; QEEG, quantitative electroencephalography; wPLI, weighted phase lag index.

**Supplementary Table S4. Sensitivity Analysis: Parsimonious multivariable logistic regression model for 90-day Futile Recanalization.**

| **Predictor** | **β Coefficient** | **SE** | **Adjusted OR (95% CI)** | **P-value** |
| --- | --- | --- | --- | --- |
| Age (per year) | 0.023 | 0.027 | 1.02 (0.97–1.08) | 0.387 |
| Baseline NIHSS (per point) | 0.097 | 0.056 | 1.10 (0.99–1.23) | 0.083 |
| Baseline blood glucose (mmol/L) | 0.277 | 0.130 | 1.32 (1.02–1.70) | 0.033 |
| Central Relative Beta Power (per SD increase) | -0.840 | 0.411 | 0.43 (0.19–0.97) | 0.041 |

Note: Evaluated in complete cases with available baseline blood glucose (N = 55; one patient with missing glucose was excluded). Model adjusted for the three primary clinical covariates. Central relative beta power remains an independent protective predictor.

*Abbreviations:* CI, confidence interval; NIHSS, National Institutes of Health Stroke Scale; OR, odds ratio; SD, standard deviation; SE, standard error.

.

**Supplementary Figure S2. Population-level power spectral density (PSD) profiles in the central Rolandic region comparing Non-FR and FR groups.**

Grand-average PSD curves (1–30 Hz) in Non-FR (n=25) vs. FR patients (n=31), showing delta-band slow-wave accumulation (1–4 Hz) and marked beta-band power suppression (13–30 Hz) in patients experiencing futile recanalization. Shaded regions represent 95% confidence intervals derived from 1000-iteration bootstrap resampling.

*Abbreviations:* FR, futile recanalization; PSD, power spectral density; QEEG, quantitative electroencephalography.

**Supplementary Figure S3. Exploratory decision curve analysis for predicting futile recanalization.**

Net benefit was estimated from leave-one-out predicted probabilities in 56 patients. Blue, red, and green curves represent the Clinical, Combined, and QEEG-only Models, respectively. Grey dashed and black horizontal lines indicate treat-all and treat-none reference strategies for hypothetical post-EVT management actions.

*Abbreviations:* DCA, decision curve analysis; EVT, endovascular treatment; LOOCV, leave-one-out cross-validation; QEEG, quantitative electroencephalography.

**Supplementary Figure S4. Calibration of Firth-penalized models using leave-one-out cross-validation.**

Panels a-c show the Clinical, Combined, and QEEG-only Models, respectively. Points represent mean predicted FR probabilities and observed FR proportions in four equally sized groups of 14 patients. Error bars indicate 95% Wilson confidence intervals for observed proportions; dashed lines indicate ideal calibration. Brier scores were calculated from individual held-out predictions. The intervals do not account for model-development uncertainty.

*Abbreviations*: FR, futile recanalization; LOOCV, leave-one-out cross-validation; QEEG, quantitative electroencephalography.

**Supplementary Figure S5. Box plots demonstrating QEEG biomarkers across ECASS hemorrhagic transformation subtypes.**

**(A)** Distribution of Delta-band brain symmetry index (BSI) across No ICH (n=40), HI (n=5), and PH groups (n=10) (Kruskal-Wallis *P* = 0.022), showing a highly significant increase specific to space-occupying parenchymal hemorrhage (PH vs. No ICH, Mann-Whitney *P* = 0.006), but not benign hemorrhagic infarction (HI vs. No ICH, Mann-Whitney *P* = 0.820). **(B)** Distribution of central relative beta power across No ICH (n=40), HI (n=5), and PH groups (n=10) (Kruskal-Wallis *P* = 0.644). Solid box borders represent medians and interquartile ranges (IQR), and individual data points are overlayed as jittered dots.

*Abbreviations:* BSI, brain symmetry index; ECASS, European Cooperative Acute Stroke Study; HI, hemorrhagic infarction; ICH, intracranial hemorrhage; PH, parenchymal hemorrhage; QEEG, quantitative electroencephalography.
